# Supplementary material for: Synapse maintenance is impacted by ATAT-2 tubulin acetyltransferase activity and the RPM-1 signaling hub
Source: eLife. 2019 Jan 18;8:e44040. doi: 10.7554/eLife.44040 (PMC6355192; doi:10.7554/eLife.44040)
Supplement: Supplementary file 1. [file elife-44040-supp1.docx]

Supplementary file 1

| **Reference Strain** | **Transgene Name** | **Figure** | **Injection Conditions^** | **Genotype*** |
| --- | --- | --- | --- | --- |
| XMN31 | *bggEx8* | 1D | P_rpm-1_RPM-1 (pCZ160), 25ng/µl  P_myo-2_RFP (pBG-24), 2ng/µl Pbluescript (pBG-49), 75ng/µl | *rpm-1; muIs32* |
| - | *2*  *transgenic lines* | 1D | P_mec-3_RPM-1::GFP (pBG-46), 20ng/µl  P_myo-2_RFP (pBG-24), 2ng/µl) pBluescript (pBG-49), 78ng/µl | *rpm-1; muIs32* |
| XMN768 | *bggEx127* | 2A, B | P_mec-3_RPM-1::GFP (pBG-46), 20ng/µl  P_mec-7_tdTOMATO (pBG-GY575), 15ng/µl P_ttx-3_GFP (pBG-40), 50ng/µl  pBluescript (pBG-49), 15ng/µl | *rpm-1; muIs32* |
| - | *4*  *transgenic lines* | 6C | P_atat-2_ATAT-2, 0.2ng/µl PCR product  P_ttx-3_RFP (pBG-41), 50ng/µl pBluescript (pBG-49), 49ng/µl | *atat-2; zdIs5* |
| - | *6*  *transgenes* | 6C | P_mec-7_ATAT-2, 0.2ng/µl PCR product  P_ttx-3_RFP (pBG-41), 50ng/µl pBluescript (pBG-49), 45ng/µl | *atat-2; zdIs5* |
| - | *6*  *transgenic lines* | 6C | P_mec-7_ATAT-2 dead, 0.2ng/µl PCR product P_ttx-3_RFP (pBG-41), 50ng/µl  pBluescript (pBG-49), 49.8ng/µl | *atat-2; zdIs5* |
| - | *3*  *transgenic lines* | 6C | P_mec-7_mCherry (pBG-GY258), 0.2ng/µl PCR product  P_ttx-3_RFP (pBG-41), 50ng/µl pBluescript (pBG-49), 49.8ng/µl | *atat-2; zdIs5* |
| XMN996 | *bggEx141* | S1 | P_mec-7_SYD-2::mScarlet (pBG-GY936), 5ng/µl P_ttx-3_RFP (pBG-41), 50ng/µl  NeoR (pBG-264), 2ng/µl  pBluescript (pBG-49), 43ng/µl | *muIs32* |
| XMN997 | *bggEx141* | S1 | P_mec-7_SYD-2::mScarlet (pBG-GY936), 5ng/µl P_ttx-3_RFP (pBG-41), 50ng/µl  NeoR (pBG-264), 2ng/µl  pBluescript (pBG-49), 43ng/µl | *rpm-1; muIs32* |
